# A study on the correlation between professional self-concept, social support and presenteeism among nurses

**DOI:** 10.3389/fpubh.2025.1631151

**Published:** 2025-09-18

**Authors:** Danqin Li, Xiaowei Liu, Shaojuan Huang, Yiyun Zeng, Jinsheng Lu

**Affiliations:** ^1^The Eighth Clinical Medical College, Guangzhou University of Chinese Medicine, Foshan, China; ^2^Department of General Medication, Foshan Hospital of Traditional Chinese Medicine, Foshan, China; ^3^Department of Orthopedics, Foshan Hospital of Traditional Chinese Medicine, Foshan, China; ^4^Department of Nursing, Foshan Hospital of Traditional Chinese Medicine, Foshan, China

**Keywords:** professional self-concept, social support, presenteeism, workload, nurses

## Abstract

**Objective:**

This study aimed to investigate the relationships between nurses' professional self-concept, social support, and presenteeism, providing insights for optimizing nursing human resource management.

**Methods:**

A cross-sectional survey was conducted using the professional self-concept scale, social support rating scale, and presenteeism scale among 520 nurses from tertiary hospitals in Guangdong Province, China. Descriptive statistics, Pearson correlation analysis, and hierarchical multiple linear regression were employed for data analysis.

**Results:**

A total of 503 valid questionnaires were collected, yielding an effective response rate of 96.73%. There was a significant negative correlation between professional self-concept and presenteeism [*r* = −0.339, 95%CI (−0.414, −0.259), *P* < 0.05]. There was also a significant negative correlation between social support and presenteeism [*r* = −0.292, 95%CI (−0.370, −0.209), *P* < 0.05]. Hierarchical multiple regression analysis showed that in Model 1, surgery, operating room, and work intensity evaluation of “relatively high” and “moderate” had obvious predictive effects on presenteeism (*P* < 0.05). In Model 2, surgery, work intensity evaluation of “relatively high” and “moderate”, and professional self-concept had obvious predictive effects on presenteeism (*P* < 0.01). In Model 3, surgery, operating room, work intensity evaluation of “relatively high” and “moderate”, professional self-concept, and social support had obvious predictive effects on presenteeism (*P* < 0.05). The *R*^2^ of Model 1, Model 2, and Model 3 were 6.6%, 16.1%, and 17.4% respectively. The results of the mediation effects test showed that perceived social support partially mediated the relationship between professional self-concept and presenteeism.

**Conclusion:**

Enhancing nurses' professional self-identity and strengthening social support may be predictive of lower levels of presenteeism, potentially contributing to improved nursing team performance and patient safety.

## 1 Introduction

With the rapid growth of healthcare demands and the acceleration of population aging, China's nursing workforce has long been under strain (1). Under the “patient-centered” care model, nurses face dual pressures from high workloads and emotional labor (2). Presenteeism, defined as attending work despite health issues that normally require rest, has become increasingly common among nurses. This is compounded by a cultural perception in Chinese workplaces where “engaging in work duties despite illness” (3, 4). The concept of presenteeism was first introduced by Professor Cooper in 1996, describing employees who continue working despite physical or mental unwellness (5). The results of a recent Meta-analysis showed that the overall estimate of the global detection rate of hidden absenteeism among nursing staff was 49.2% (6). Nurses are recognized as a high-risk group for work-related stress. They often face time pressure, role conflict, and overload (7). The occurrence of hidden absenteeism not only negatively affects nurses' personal health, quality of life, and well-being index, but also professionally causes a decrease in work motivation, mental acuity, and communication skills, as well as possible adverse consequences for patient safety and quality of care (8). Due to the influence of traditional thinking and culture, China still largely promotes the idea of “working with illness” and recognizes it as a form of professionalism. However, Lui et al. (9) that the seasonal influenza-associated surge in hospital occupancy in Hong Kong resulted in an implicit absenteeism productivity loss for nurses in three hospitals of ~US$24,096/year, which is one of the high costs reported in global healthcare workforce studies. Another survey of employees from different industries in China and the UK showed that Chinese employees exhibited higher levels of hidden absenteeism than UK employees (10). Therefore, it is important to conduct a study on hidden absenteeism in the nurse population based on the cultural context of China.

Professional self-concept plays an important central and driving role in one's career choice and professional development, and is a reflection of self-concept in professional choice and professional development (11). It comes from an individual's knowledge of self and of the profession, and on this basis, it forms an individual's attitude toward the profession, sense of professional responsibility, professional ideals, professional ethics and professional values (12). The formation and development of professional self-concept occurs throughout a person's life, develops with physiological and psychological growth, and develops gradually based on the individual's observation and experience of the profession, and the family, school, and society also have a certain influence on the formation of the individual's professional self-concept (13).

Perceived social support refers to a psychological experience in which an individual subjectively feels that he or she is understood, respected, and able to receive help and support from others in the process of interacting with others (14). Research has shown that those individuals with higher perceptions of social support tend to be more likely to gain emotional understanding and help from their surroundings, thus accumulating more positive psychological resources (15). A related study found an association between higher levels of perceived social support and higher work efficiency as well as lower attendance list behavior (16). According to conservation of resources (COR) theory, when individuals perceive a threat to their resources (e.g., time, energy, self-efficacy, or social support), they feel stress and may adopt maladaptive behaviors such as presenteeism as a compensatory strategy (17). In nursing, a weak occupational self-concept may imply a depletion of personal resources (e.g., confidence, professional identity), whereas lower social support may reflect a lack of external resources. Both conditions may increase the likelihood that presenteeism will occur. However, current research has under-explored the relationship between occupational self-concept, social support, and presenteeism.

**Figure 1:**
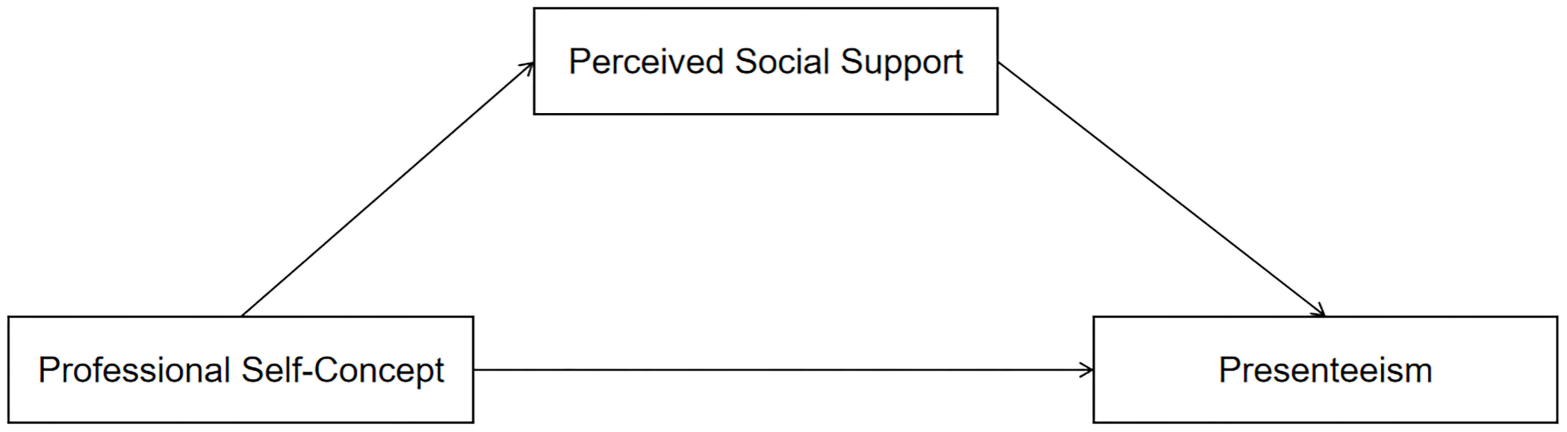
Theoretical model.

Based on the above theoretical model, this study took nurses' professional self-concept and social support as independent variables and hidden absenteeism as dependent variable and proposed the following hypotheses: hypothesis 1: nurses' professional self-concept is negatively correlated with hidden absenteeism; hypothesis 2: nurses' perceived social support is negatively correlated with hidden absenteeism; and hypothesis 3: social support mediates the relationship between professional self-concept and hidden absenteeism (Figure 1).

This study is the for the first time, the effects of professional self-concept and perceived social support on presenteeism were examined simultaneously in a group of Chinese nurses to provide a reference basis for improving nursing quality and ensuring patient safety.

## 2 Research methods

### 2.1 Study participants

A cross-sectional study design was utilized. Convenience sampling was used to recruit 520 clinical nurses from tertiary hospitals in Guangdong Province, China, between September 2024 and February 2025.

According to Kendall's sample size calculation guideline, survey samples should be at least five to ten times the number of independent variables (18). This study included 68 explanatory variables. There are 20 variables in the general demographic questionnaire, 30 variables in the professional self-concept scale, 6 variables in the presenteeism scale, and 12 variables in the perceived social support scale. Based on this principle, the estimated required sample size ranged from 340 to 680 participants. Considering a 20% invalid questionnaire rate, we determined a final sample size of 520 participants. This calculation demonstrates that our study's sample size was sufficient.

Inclusion criteria: (1) possession of a valid nurse qualification certificate; (2) over one year of clinical work experience in hospital departments; (3) active engagement in clinical nursing during the study period; (4) employment at a tertiary hospital during the study period; (5) informed consent and voluntary participation.

Exclusion criteria: (1) nurses rehired after retirement; (2) nurses from external hospitals undergoing training or internship.

Ethical approval for this study was waived by the Ethics Committee of Foshan Hospital of Traditional Chinese Medicine in accordance with institutional and national guidelines. Written informed consent was obtained from all participants.

### 2.2 Research tools

#### 2.2.1 General demographic questionnaire

The researchers developed a basic information questionnaire for study participants by reviewing relevant domestic and international literature. The questionnaire collected data on the following aspects: gender, age, years of nursing experience, education level, professional title, marital status, work department, presence of children, family life stage, average monthly household income, and average number of night shifts per month.

#### 2.2.2 Professional self-concept scale

The Nursing Professional Self-Concept Scale developed by Arthur was used to measure the research subjects (19). The scale consists of 30 items across 5 domains: management ability (4 items), flexibility (6 items), professional skills (5 items), satisfaction (9 items), and communication ability (7 items). Among these, 7 items are reverse-scored. Each item uses a 4-point Likert scale, with positive items scored positively and negative items scored negatively. For all positive items, 1 indicates the most negative professional self-attitude, and 4 indicates the most positive professional self-attitude. The reverse applies to negative items. Higher scores indicate stronger professional self-concept. The total score ranges from 30 to 120, with scores above 75 indicating a positive professional self-attitude. The scale was officially translated and revised by Chinese scholar Yang (20). Test Cronbach's α coefficient of the Chinese version of the scale showed 0.84. Exploratory and validation analyses showed that the scale had high reliability and validity.

#### 2.2.3 Presenteeism scale

The Stanford Presenteeism Scale-6 (SPS-6) developed by Koopman et al. (21) was used to measure the research subjects. The scale consists of 6 items across 2 domains: work impairment (4 items) and work energy (2 items). Each item uses a 5-point Likert scale, ranging from 1 (not at all) to 5 (completely). Items 5 and 6 are reverse-scored. The total score ranges from 6 to 30. Higher scores indicate greater productivity loss due to presenteeism and more severe presenteeism. The scale was officially translated and revised by Chinese scholar Zhao (22). The test Cronbach'sa coefficient for the Chinese version of the scale showed 0.79. Exploratory and validation analyses showed that the reliability and validity of the scale were high.

#### 2.2.4 Perceived social support scale

The perceived social support scale developed by Zimet et al. (23) to measure the study subjects. The scale consists of 12 items across 3 domains: family support (4 items), friend support (4 items), and other support (4 items). Each item uses a 7-point Likert scale, ranging from 1 (strongly disagree) to 7 (strongly agree). Higher total scores indicate that individuals are more likely to receive support and perceive more social support. The scale was officially translated and revised by the Chinese scholar Huang (24). Test Cronbach's α coefficient of the Chinese version of the scale showed 0.85. Exploratory and validation analyses showed high reliability and validity of the scale.

### 2.3 Data collection

The study was conducted in tertiary hospitals across Guangdong Province, China, from September 2024 to March 2025. Eligible nurses meeting the inclusion criteria were recruited. To ensure data quality, data collectors received standardized training. Electronic questionnaires were distributed and collected via the online platform (Wenjuanxing). Technical Measures Taken to Ensure Data Validity: We set a unique IP address limit to prevent multiple submissions from the same device and to ensure that each participant could only complete the survey once. We also set a reasonable window of time (30 minutes) for survey completion and automatically save responses to avoid hasty or incomplete submissions while allowing enough time for thoughtful responses. Additionally, we utilized the platform's built-in data encryption and anonymization features to protect participant information and confirm that no identifying data (such as name or contact information) was collected, which also helped to maintain the authenticity of the responses by reducing social desirability bias.

### 2.4 Statistical analysis

Data were statistically analyzed using SPSS 23.0. Normality of the data was assessed using the Kolmogorov-Smirnov test. For variables that passed the normality test (*P* > 0.05), descriptive statistics were presented as mean ± standard deviation (SD), and inferential analyses for group differences (e.g., across demographic variables) were conducted using *t*-tests (for two independent groups) or one-way analysis of variance (ANOVA; for three or more groups). For variables that violated the normality assumption (*P* ≤ 0.05), descriptive statistics were expressed as median and interquartile range (IQR), and non-parametric tests (Mann–Whitney *U* test for two groups or Kruskal–Wallis *H* test for multiple groups) were applied to examine group differences. Professional self-concept, social support, and presenteeism total scores followed a normal distribution, so pearson correlation analysis was used to explore the correlations among the three variables.

A statistically significant difference in general demographic information with implicit absenteeism as the dependent variable was used as the independent variable, and professional self-concept and social support were also added to test for covariance. Subsequently, we performed hierarchical linear regression: model 1 incorporated variables with statistically significant differences in hidden absenteeism on general demographic information and no covariance as independent variables; model 2 added adding professional self-concept to model 1; and model 3 incorporated perceived social support to model 2. In addition, the model was plotted using Mplus 8.1 to test whether perceived social support was a mediating variable between clinical professional self-concept and Presenteeism, and the Bootstrap method was used to analyze the mediating effect. A *P*-value < 0.05 was considered statistically significant.

## 3 Results

### 3.1 Demographic characteristics

A total of 520 questionnaires were distributed. Eight nurses discontinued their participation due to emergencies, and their incomplete responses were deemed invalid. Six questionnaires with identical answers completed in unusually short time frames, along with three containing logical inconsistencies, were also invalidated. Ultimately, 503 valid questionnaires were collected, yielding an effective response rate of 96.73%. The demographic data for the 503 nurses are presented in Table 1.

**Table 1:** Demographic characteristics.

**Variable**	**Stratification**	**Number of cases (*n*)**	**Percentage (%)**
Gender	Male	37	7.54
	Female	466	92.46
Age	< 25	46	9.13
	25–29	123	24.40
	30–34	115	23.02
	35–39	74	14.68
	≥40	145	28.77
Ethnic group	Han	494	98.21
	Ethnic minorities	9	1.79
Marital status	Unmarried	171	33.93
	Married	327	65.08
	Divorced	5	0.99
Presence of children	None	204	40.48
	One child	134	26.79
	Two children	160	31.75
	Three or more children	5	0.99
Family children stage	No children	207	41.07
	Infant	101	20.04
	Primary school	116	23.21
	Junior & senior high school and above	79	15.67
Educational level	Junior college	58	11.51
	Bachelor's degree or above	445	88.49
Years of nursing experience	1–5 years	135	26.79
	6–10 years	128	25.60
	11–15 years	59	11.71
	≥16 years	181	35.91
Professional title	None	5	0.99
	Nurse	89	17.66
	Nurse-in-charge	177	35.32
	Supervisor nurse	177	35.12
	Deputy director nurse or above	56	11.11
Administrative position	None	452	89.88
	Yes	51	10.12
Employment status	Non-permanent	345	68.65
	Permanent	158	31.35
Department	Internal medicine	159	31.55
	Surgery	224	44.44
	Operating room	65	13.10
	Others	55	10.91
Did your job require night shifts in the past year?	Yes	463	92.06
	No	40	7.94
Number of night shifts per month	≤ 5	476	94.64
	>5	27	5.36
Weekly working hours	≤ 40 h	386	76.79
	>40 h	117	23.21
Evaluation of current work intensity	Very high	96	19.05
	Relatively high	231	46.03
	Moderate	168	33.33
	Relatively low	7	1.39
	Very low	1	0.20
Average monthly income	< 6,000 CNY	64	12.70
	6,001–8,000 CNY	105	20.83
	8,001–10,000 CNY	164	32.54
	>10,000 CNY	170	33.93
Satisfaction with effort and compensation	Very dissatisfied	19	3.77
	Dissatisfied	60	11.90
	Neutral	269	53.37
	Satisfied	136	27.18
	Very satisfied	19	3.77
Family's average monthly income	≤ 4,000 yuan	47	9.33
	4,001–6,000 CNY	78	15.67
	6,001–8,000 CNY	99	19.64
	8,001–10,000 CNY	98	19.44
	>10,000 CNY	181	35.91
Overall evaluation of family economic status	Very tight	40	7.94
	Relatively tight	131	25.99
	Moderate	297	58.93
	Relatively affluent	34	6.75
	Very affluent	2	0.40

### 3.2 Scores of nurses' professional self-concept, perceived social support, and presenteeism

The total score for nurses' professional self-concept was 86.79 ± 11.01 points. Dimension scores ranked from highest to lowest were communication skills, professional skills, management abilities, and adaptability. The total perceived social support score was 63.53 ± 12.12 points, with dimensions ranked as family support, friend support, and other support. The total presenteeism score was 15.14 ± 4.58 points, with dimensions ranked as work limitations and work energy. Detailed scores are shown in Table 2.

**Table 2:** Differences in nurses' professional self-concept across demographic characteristics.

**Variable**	**Scoring range**	**Items (Mean ±SD)**	**Total points (Mean ±SD)**
**Professional self-concept scale**	30–120	2.89 ± 0.37	86.79 ± 11.01
Management skills	4–16	2.86 ± 0.41	11.43 ± 1.64
Flexibility	7–28	2.77 ± 0.51	16.63 ± 3.04
Professional competence	5–20	2.93 ± 0.46	14.65 ± 2.32
Job satisfaction	5–20	2.92 ± 0.39	26.30 ± 3.48
Communication skills	5–20	2.96 ± 0.37	17.77 ± 2.24
**Perceived social support scale**	12–84	5.29 ± 1.01	63.53 ± 12.12
Family support	4–28	5.37 ± 1.13	21.48 ± 4.53
Friend support	4-−28	5.29 ± 1.04	21.15 ± 4.17
Other support	4–28	5.23 ± 1.08	20.90 ± 4.33
**Presenteeism scale**	6–30	2.05 ± 0.76	15.14 ± 4.58
Work limitations	4–20	2.20 ± 1.02	8.80 ± 4.10
Work energy	2–10	3.17 ± 1.18	6.34 ± 2.36

### 3.3 Differences in nurses' professional self-concept across demographic characteristics

Table 3 presents statistically significant differences in professional self-concept across key demographic variables (*P* < 0.05), including age, marital status, parental status, child-rearing stage, nursing experience, professional title, administrative role, employment type, department, night shift requirement, weekly working hours, monthly income, satisfaction with pay-effort ratio, and overall assessment of family economic status. High-scoring groups included nurses aged 40+, married, with children (especially high school-aged+), holding administrative/professional titles, permanent positions, working in operating rooms, earning high incomes, reporting affluent family finances, and expressing job satisfaction. Lower scores were seen among younger, unmarried/divorced, childless, non-administrative/untitled, contract, internal medicine/surgical, low-income, financially strained, and nurses reporting job dissatisfaction.

**Table 3:** Differences in nurses' professional self-concept across demographic characteristics.

**Variable**	**Stratification**	**Number of cases (n)**	**Percentage (%)**	**Mean ±SD**	***t*/*F***	***P***	**Cohen's *d*/omega-squared (95% CI)**
Gender	Male	37	7.54	87.57 ± 10.46	0.446	0.655	0.076 (−0.259, 0.411)
	Female	466	92.46	86.73 ± 11.06			
Age	< 25	46	9.13	82.13 ± 6.14	13.676	0.000^*^	0.099 (0.050, 0.145)
	25–29	123	24.40	84.96 ± 9.18			
	30–34	115	23.02	85.19 ± 11.75			
	35–39	74	14.68	84.86 ± 10.99			
	≥40	145	28.77	92.07 ± 11.26			
Ethnic group	Han	494	98.21	86.71 ± 11.05	−1.220	0.223	−0.410 (−1.070, 0.250)
	Ethnic Minorities	9	1.79	91.22 ± 7.89			
Marital status	Unmarried	171	33.93	84.77 ± 9.21	7.164	0.001^*^	0.024 (0.001, 0.056)
	Married	327	65.08	88.00 ± 11.64			
	Divorced	5	0.99	76.60 ± 11.95			
Presence of children	None	204	40.48	84.77 ± 9.11	4.444	0.004^*^	0.020 (−0.003, 0.049)
	One child	134	26.79	87.35 ± 12.10			
	Two children	160	31.75	88.75 ± 11.93			
	Three or more children	5	0.99	91.20 ± 8.44			
Family children stage	No children	207	41.07	84.66 ± 8.99	8.647	0.001^*^	0.044 (0.010, 0.081)
	Infant	101	20.04	85.69 ± 12.45			
	Primary school	116	23.21	88.45 ± 11.37			
	Junior & senior high school and above	79	15.67	91.33 ± 11.77			
Educational level	Junior college	58	11.51	85.55 ± 11.49	−0.910	0.363	−0.127 (−0.401, 0.147)
	Bachelor's degree or above	445	88.49	86.95 ± 10.95			
Years of nursing experience	1–5 years	135	26.79	84.07 ± 8.48	14.273	0.001^*^	0.073 (0.030, 0.118)
	6–10 years	128	25.60	85.31 ± 11.22			
	11–15 years	59	11.71	83.75 ± 11.15			
	≥16 years	181	35.91	90.85 ± 11.35			
Professional title	None	5	0.99	84.80 ± 3.70	11.883	0.001^*^	0.080 (0.033, 0.124)
	Nurse	89	17.66	83.45 ± 9.42			
	Nurse-in-charge	177	35.32	85.14 ± 10.43			
	Supervisor nurse	177	35.12	87.64 ± 11.34			
	Deputy director nurse or above	56	11.11	94.96 ± 10.42			
Administrative position	None	452	89.88	86.02 ± 10.78	−4.781	0.001^*^	−0.706 (−0.999, −0.413)
	Yes	51	10.12	93.63 ± 10.71			
Employment status	Non-permanent	345	68.65	84.50 ± 10.22	−7.242	0.001^*^	−0.696 (−0.888, −0.502)
	Permanent	158	31.35	91.79 ± 11.04			
Department	Internal medicine	159	31.55	86.22 ± 11.52	2.960	0.032^*^	0.012 (−0.006, 0.036)
	Surgery	224	44.44	86.02 ± 10.34			
	Operating room	65	13.10	90.45 ± 11.23			
	Others	55	10.91	87.24 ± 11.31			
Did your job require night shifts in the past year?	Yes	463	92.06	86.05 ± 10.69	−5.294	0.001^*^	−0.872 (−1.200, −0.545)
	No	40	7.94	95.40 ± 11.05			
Number of night shifts per month	≤ 5	476	94.64	86.69 ± 11.03	−0.857	0.392	−0.170 (−0.557, 0.218)
	>5	27	5.36	88.56 ± 10.60			
Weekly working hours	≤ 40 h	386	76.79	87.32 ± 11.11	1.984	0.048^*^	0.209 (0.002, 0.417)
	>40 h	117	23.21	85.03 ± 10.51			
Evaluation of current work intensity	Very high	96	19.05	85.29 ± 12.29	0.865	0.485	−0.001 (−0.008, 0.012)
	Relatively high	231	46.03	86.88 ± 11.05			
	Moderate	168	33.33	87.51 ± 10.13			
	Relatively low	7	1.39	88.43 ± 11.67			
	Very low	1	0.20	77.00			
Average monthly income	< 6,000 CNY	64	12.70	83.70 ± 8.36	2.961	0.032	0.012 (−0.006, 0.036)
	6,001–8,000 CNY	105	20.83	85.97 ± 10.32			
	8,001–0,000 CNY	164	32.54	88.30 ± 11.67			
	>10,000 CNY	170	33.93	86.99 ± 11.44			
Satisfaction with effort and compensation	Very dissatisfied	19	3.77	81.05 ± 15.60	12.030	0.001^*^	0.088 (0.041, 0.132)
	Dissatisfied	60	11.90	83.27 ± 10.77			
	Neutral	269	53.37	85.90 ± 10.49			
	Satisfied	136	27.18	89.13 ± 9.84			
	Very satisfied	19	3.77	99.42 ± 9.63			
Family's average monthly income	≤ 4,000 CNY	47	9.33	83.23 ± 10.97	3.074	0.016^*^	0.016 (−0.007, 0.042)
	4,001–6,000 CNY	78	15.67	85.44 ± 10.31			
	6,001–8,000 CNY	99	19.64	86.57 ± 11.03			
	8,001–10,000 CNY	98	19.44	86.19 ± 10.47			
	>10,000 CNY	181	35.91	88.74 ± 11.33			
Overall evaluation of family economic status	Very tight	40	7.94	81.18 ± 12.58	3.608	0.007^*^	0.020 (−0.006, 0.048)
	Relatively tight	131	25.99	86.51 ± 10.83			
	Moderate	297	58.93	87.28 ± 10.76			
	Relatively affluent	34	6.75	90.06 ± 10.37			
	Very affluent	2	0.40	88.50 ± 0.71			

^*^P < 0.05.

### 3.4 Differences in nurses' perceived social support across demographic characteristics

Table 4 indicates statistically significant differences (*P* < 0.05) in perceived social support across age, nursing experience, technical title, administrative role, employment type, night shift requirement, weekly work hours, satisfaction with pay-effort ratio, family income per capita, and overall assessment of family economic status. High-scoring groups included nurses aged < 25 or ≥40, holding administrative/professional titles (≥deputy director nurse or Above), permanent positions, non-night shifts, ≤ 40 h/week, high family incomes (>10,000 CNY), “very affluent” financial status, and “very satisfied” with effort-reward balance. Lower scores were observed among 35–39-year-olds, registered/practitioner nurses, contract/administrative staff, night-shift workers, >40 h/week, ≤ 4,000 CNY family income, “very tight” finances, and “very dissatisfied” nurses.

**Table 4:** Differences in nurses' social support across demographic characteristics.

**Variable**	**Stratification**	**Number of cases (n)**	**Percentage (%)**	**Mean ±SD**	***t*/*F***	***P***	**Cohen's *d* (95%CI)**
Gender	Male	37	7.54	66.24 ± 13.14	1.418	0.157	0.242 (−0.093, 0.577)
	Female	466	92.46	63.31 ± 12.03			
Age	< 25	46	9.13	64.48 ± 9.27	4.296	0.002^*^	0.026 (−0.003, 0.055)
	25–29	123	24.40	64.20 ± 11.51			
	30–34	115	23.02	62.02 ± 13.15			
	35–39	74	14.68	59.42 ± 11.58			
	≥40	145	28.77	65.95 ± 12.30			
Ethnic group	Han	494	98.21	63.46 ± 12.16	−0.978	0.328	−0.329 (−0.988, 0.331)
	Ethnic minorities	9	1.79	67.44 ± 9.84			
Marital status	Unmarried	171	33.93	63.70 ± 11.06	0.037	0.963	−0.004 (−0.004, −0.002)
	Married	327	65.08	63.45 ± 12.63			
	Divorced	5	0.99	62.60 ± 15.29			
Presence of children	None	204	40.48	63.69 ± 11.27	0.443	0.722	−0.003 (−0.006, 0.006)
	One child	134	26.79	62.69 ± 13.69			
	Two children	160	31.75	64.11 ± 11.91			
	Three or more children	5	0.99	60.60 ± 8.88			
Family children stage	No children	207	41.07	63.76 ± 10.99	0.748	0.524	−0.002 (−0.006, 0.011)
	Infant	101	20.04	61.94 ± 13.47			
	Primary school	116	23.21	64.04 ± 11.87			
	Junior & senior High school and above	79	15.67	64.18 ± 13.49			
Educational level	Junior college	58	11.51	61.93 ± 13.44	−1.066	0.287	−0.149 (−0.243, 0.125)
	Bachelor's degree or above	445	88.49	63.73 ± 11.94			
Years of nursing experience	1–5 years	135	26.79	64.75 ± 11.00	2.746	0.042^*^	0.010 (−0.006, 0.034)
	6–10 years	128	25.60	61.34 ± 12.46			
	11–15 years	59	11.71	61.97 ± 12.56			
	≥16 years	181	35.91	64.67 ± 12.36			
Professional title	None	5	0.99	65.60 ± 4.34	3.511	0.008^*^	0.020 (−0.006, 0.047)
	Nurse	89	17.66	63.35 ± 12.87			
	Nurse-in-charge	177	35.32	62.75 ± 11.52			
	Supervisor nurse	177	35.12	62.59 ± 12.54			
	Deputy director nurse or above	56	11.11	69.15 ± 10.58			
Administrative position	None	452	89.88	63.02 ± 12.13	−2.837	0.005^*^	−0.419 (−0.709, −0.128)
	Yes	51	10.12	68.06 ± 11.14			
Employment status	Non-permanent	345	68.65	62.53 ± 12.14	−2.734	0.006^*^	−0.263 (−0.451, −0.074)
	Permanent	158	31.35	65.70 ± 11.83			
Department	Internal medicine	159	31.55	63.08 ± 12.10	1.931	0.124	0.006 (−0.006, 0.036)
	Surgery	224	44.44	62.62 ± 12.33			
	Operating room	65	13.10	66.02 ± 12.07			
	Others	55	10.91	65.56 ± 10.99			
Did your job require night shifts in the past year?	Yes	463	92.06	62.94 ± 12.24	−3.759	0.001^*^	−0.620 (−0.944, −0.294)
	No	40	7.94	70.35 ± 8.02			
Number of night shifts per month	≤ 5	476	94.64	63.44 ± 12.13	−0.681	0.496	−0.135 (−0.523, 0.253)
	>5	27	5.36	65.07 ± 12.17			
Weekly working hours	≤ 40 h	386	76.79	64.31 ± 11.86	2.633	0.009^*^	0.278 (0.070, 0.485)
	>40 h	117	23.21	60.96 ± 12.65			
Evaluation of current work Intensity	Very high	96	19.05	60.80 ± 14.05	2.030	0.089	0.008 (−0.008, 0.29)
	Relatively high	231	46.03	63.54 ± 12.33			
	Moderate	168	33.33	64.86 ± 10.35			
	Relatively low	7	1.39	67.29 ± 12.61			
	Very low	1	0.20	72.00			
Average monthly income	< 6,000 CNY	64	12.70	61.59 ± 10.03	0.941	0.421	0.000 (−0.006, 0.014)
	6,001–8,000 CNY	105	20.83	62.80 ± 12.37			
	8,001–10,000 CNY	164	32.54	64.16 ± 12.36			
	>10,000 CNY	170	33.93	64.09 ± 12.44			
Satisfaction with effort and compensation	Very dissatisfied	19	3.77	55.47 ± 18.69	13.548	0.001^*^	0.091 (0.042, 0.137)
	Dissatisfied	60	11.90	58.27 ± 10.32			
	Neutral	269	53.37	62.65 ± 11.78			
	Satisfied	136	27.18	67.14 ± 10.80			
	Very satisfied	19	3.77	74.68 ± 7.51			
Family's average monthly income	≤ 4000 CNY	47	9.33	63.81 ± 11.12	2.739	0.028^*^	0.014 (−0.008, 0.038)
	4,001–6,000 CNY	78	15.67	61.35 ± 11.72			
	6,001–8,000 CNY	99	19.64	62.74 ± 12.20			
	8,001–10,000 CNY	98	19.44	61.86 ± 11.62			
	>10,000 CNY	181	35.91	65.73 ± 12.52			
Overall evaluation of family economic status	Very tight	40	7.94	56.68 ± 15.12	9.215	0.001^*^	0.061 (0.019, 0.102)
	Relatively tight	131	25.99	60.45 ± 10.76			
	Moderate	297	58.93	65.16 ± 11.71			
	Relatively affluent	34	6.75	68.18 ± 11.22			
	Very affluent	2	0.40	78.50 ± 7.78			

^*^P < 0.05.

### 3.5 Differences in nurses' presenteeism across demographic characteristics

Table 5 shows statistically significant differences (*P* < 0.05) in terms of presenteeism, broken down by department, night shift requirement, perceived work intensity, satisfaction with the pay-effort ratio and an overall assessment of family economic status. High-scoring groups included internal medicine nurses, night-shift workers, those reporting “very high” workload, “very dissatisfied” effort-reward satisfaction, and “very tight” family finances. Lower scores were seen among surgical/operating room nurses, non-night-shift staff, nurses with lower workload ratings, “very satisfied” effort-reward perceptions, and “very affluent” families.

**Table 5:** Differences in nurses' presenteeism across demographic characteristics.

**Variable**	**Stratification**	**Number of cases (n)**	**Percentage (%)**	**Mean ±SD**	***t*/*F***	***P***	**Cohen's *d* (95% CI)**
Gender	Male	37	7.54	14.46 ± 4.23	−0.938	0.349	−0.160 (−0.495, 0.175)
	Female	466	92.46	15.19 ± 4.61			
Age	< 25	46	9.13	15.13 ± 2.93	0.674	0.610	−0.003 (−0.008, 0.008)
	25–29	123	24.40	15.18 ± 4.24			
	30–34	115	23.02	14.85 ± 4.25			
	35–39	74	14.68	15.89 ± 5.90			
	≥40	145	28.77	14.95 ± 4.78			
Ethnic group	Han	494	98.21	15.11 ± 4.59	−1.231	0.219	−0.414 (−1.073, 0.246)
	Ethnic minorities	9	1.79	17.00 ± 3.91			
Marital status	Unmarried	171	33.93	15.05 ± 4.06	0.361	0.697	−0.003 (−0.004, 0.008)
	Married	327	65.08	15.16 ± 4.77			
	Divorced	5	0.99	16.80 ± 8.67			
Presence of children	None	204	40.48	15.22 ± 4.11	0.268	0.849	−0.004 (−0.006, 0.002)
	One child	134	26.79	14.87 ± 4.81			
	Two children	160	31.75	15.29 ± 4.96			
	Three or more children	5	0.99	14.40 ± 4.78			
Family children stage	No children	207	41.07	15.28 ± 4.16	0.270	0.847	−0.004 (−0.006, 0.002)
	Infant	101	20.04	14.86 ± 4.69			
	Primary school	116	23.21	15.27 ± 5.19			
	Junior & senior high school and above	79	15.67	14.94 ± 4.60			
Educational level	Junior college	58	11.51	15.47 ± 3.75	0.577	0.564	0.080 (−0.193, 0.354)
	Bachelor's degree or above	445	88.49	15.10 ± 4.68			
Years of nursing experience	1–5 years	135	26.79	15.03 ± 3.82	0.727	0.536	−0.002 (−0.006, 0.011)
	6–10 years	128	25.60	15.01 ± 4.15			
	11–15 years	59	11.71	15.97 ± 5.40			
	≥16 years	181	35.91	15.04 ± 5.08			
Professional title	None	5	0.99	13.60 ± 3.21	1.095	0.358	0.001 (−0.008, 0.016)
	Nurse	89	17.66	14.48 ± 3.85			
	Nurse-in-charge	177	35.32	15.47 ± 4.20			
	Supervisor nurse	177	35.12	15.34 ± 5.17			
	Deputy director Nurse or above	56	11.11	14.62 ± 4.86			
Administrative position	None	452	89.88	15.17 ± 4.57	0.487	0.627	0.072(−0.218, 0.361)
	Yes	51	10.12	14.84 ± 4.67			
Employment status	Non-permanent	345	68.65	15.32 ± 4.50	1.322	0.187	0.127 (−0.061, 0.315)
	Permanent	158	31.35	14.74 ± 4.73			
Department	Internal medicine	159	31.55	16.03 ± 4.54	3.536	0.015^*^	0.015 (−0.005, 0.041)
	Surgery	224	44.44	14.86 ± 4.61			
	Operating room	65	13.10	14.06 ± 4.26			
	Others	55	10.91	15.00 ± 4.64			
Did your job require night shifts in the past year?	Yes	463	92.06	15.29 ± 4.54	2.516	0.012^*^	0.415 (0.090, 0.739)
	No	40	7.94	13.40 ± 4.79			
Number of night shifts per month	≤ 5	476	94.64	15.21 ± 4.59	1.416	0.157	0.280 (−0.108, 0.667)
	>5	27	5.36	13.93 ± 4.39			
Weekly working hours	≤ 40 h	386	76.79	15.08 ± 4.46	−0.523	0.601	−0.055 (−0.262, 0.152)
	>40 h	117	23.21	15.33 ± 4.97			
Evaluation of current work intensity	Very high	96	19.05	17.33 ± 5.29	7.593	0.001^*^	0.050 (0.012, 0.087)
	Relatively high	231	46.03	14.84 ± 4.38			
	Moderate	168	33.33	14.36 ± 4.04			
	Relatively low	7	1.39	13.43 ± 4.54			
	Very low	1	0.20	16.00			
Average monthly income	< 6,000 CNY	64	12.70	14.69 ± 3.53	1.725	0.161	0.012 (−0.006, 0.036)
	6,001–8,000 CNY	105	20.83	15.18 ± 4.57			
	8,001–10,000 CNY	164	32.54	15.75 ± 5.16			
	>10,000 CNY	170	33.93	14.69 ± 4.31			
Satisfaction with effort and compensation	Very dissatisfied	19	3.77	16.95 ± 5.64	14.369	0.001^*^	0.096 (0.046, 0.143)
	Dissatisfied	60	11.90	17.80 ± 4.72			
	Neutral	269	53.37	15.41 ± 4.55			
	Satisfied	136	27.18	13.74 ± 3.59			
	Very satisfied	19	3.77	11.05 ± 3.81			
Family's average monthly income	≤ 4,000 CNY	47	9.33	15.83 ± 4.28	0.598	0.664	−0.003 (−0.008, 0.007)
	4,001–6,000 CNY	78	15.67	15.41 ± 4.79			
	6,001–8,000 CNY	99	19.64	15.28 ± 4.74			
	8,001–10,000 CNY	98	19.44	15.05 ± 4.28			
	>10,000 CNY	181	35.91	14.81 ± 4.65			
Overall evaluation of family economic status	Very tight	40	7.94	17.48 ± 4.63	4.803	0.001^*^	0.029 (−0.001, 0.061)
	Relatively tight	131	25.99	15.85 ± 4.07			
	Moderate	297	58.93	14.55 ± 4.63			
	Relatively affluent	34	6.75	14.82 ± 4.96			
	Very affluent	2	0.40	14.50 ± 0.71			

^*^P < 0.05.

### 3.6 Correlations between professional self-concept, perceived social support, and presenteeism

Table 6 shows that total professional self-concept scores were negatively correlated with total presenteeism scores [*r* = −0.339, 95%CI (−0.414, −0.259), *P* < 0.05]. Management abilities [*r* = −0.318, 95%CI (−0.394, 0.237), *P* < 0.05], adaptability [*r* = −0.252, 95%CI (−0.332, −0.148), *P* < 0.05], professional skills [*r* = −0.312, 95%CI (−0.389, −0.231), *P* < 0.05], job satisfaction [*r* = −0.294, 95% (−0.371, −0.211), *P* < 0.05], communication skills [*r* = −0.315, 95% (−0.391, −0.233), *P* < 0.05], and total perceived social support [*r* = −0.292, 95% (−0.370, −0.209), *P* < 0.05] were also negatively correlated with presenteeism. Family support [*r* = −0.239, 95%CI (−0.320, −0.155), *P* < 0.05], friend support [*r* = −0.270, 95%CI (−0.349, −0.187), *P* < 0.05], and other support [*r* = −0.307, 95% (−0.307, −0.383, −0.255), *P* < 0.05].

**Table 6:** Correlations between professional self-concept, social support, and presenteeism, *r* (95% CI).

**Variable**	**Professional self-concept scale, *r* (95% CI)**	**Management skills, *r* (95% CI)**	**Flexibility, *r* (95% CI)>**	**Professional competence, *r* (95% CI)**	**Job satisfaction, *r* (95% CI)**	**Communication skills, *r* (95% CI)**	**Perceived social support scale, *r* (95% CI)**	**Family support, *r* (95% CI)**	**Friend support, *r* (95% CI)**	**Other support, *r* (95% CI)**	**Presenteeism scale, *r* (95% CI)**	**Work limitations, *r* (95% CI)**	**Work energy, *r* (95% CI)**
**Professional self-concept scale**	1												
Management Skills	0.747^*^ (0.705, 0.783)	1											
Flexibility	0.872^*^ (0.850, 0.982)	0.534^*^ (0.469, 0.594)	1										
Professional Competence	0.905^*^ (0.887, 0.919)	0.664^*^ (0.612, 0.710)	0.755^*^ (0.714, 0.790)	1									
Job satisfaction	0.904^*^ (0.887, 0.919)	0.558^*^ (0.494, 0.615)	0.743^*^ (0.700, 0.779)	0.753^*^ (0.713, 0.789)	1								
Communication skills	0.842^*^ (0.814, 0.865)	0.659^*^ (0.606, 0.706)	0.604^*^ (0.545, 0.656)	0.729^*^ (0.685, 0.768)	0.695^*^ (0.647, 0.738)	1							
**Perceived social support scale**	0.513^*^ (0.445, 0.574)	0.396^*^ (0.319, 0.467)	0.397^*^ (0.320, 0.468)	0.481^*^ (0.411, 0.545)	0.493^*^ (0.424, 0.556)	0.426^*^ (0.351, 0.494)	1						
Family support	0.451^*^ (0.378, 0.517)	0.348^*^ (0.269, 0.422)	0.342^*^ (0.262, 0.417)	0.449^*^ (0.376, 0.516)	0.425^*^ (0.350, 0.493)	0.371^*^ (0.293, 0.444)	0.920^*^ (0.906, 0.933)	1					
Friend support	0.460^*^ (0.287, 0.526)	0.355^*^ (0.276, 0.429)	0.367^*^ (0.288, 0.440)	0.416^*^ (0.341, 0.486)	0.450^*^ (0.377, 0.517)	0.371^*^ (0.293, 0.444)	0.951^*^ (0.942, 0.959)	0.824^*^ (0.794, 0.850)	1				
Other support	0.520^*^ (0.453, 0.581)	0.403^*^ (0.326, 0.473)	0.400^*^ (0.323, 0.470)	0.476^*^ (0.405, 0.541)	0.504^*^ (0.435, 0.566)	0.445^*^ (0.372, 0.512)	0.920^*^ (0.906, 0.933)	0.737^*^ (0.694, 0.774)	0.836^*^ (0.807, 0.860)	1			
**Presenteeism scale**	−0.339^*^ (−0.414, −0.259)	−0.318^*^ (−0.394, −0.237)	−0.252^*^ (−0.332, −0.168)	−0.312^*^ (−0.389, −0.231)	−0.294^*^ (−0.371, −0.211)	−0.315^*^ (−0.391, −0.233)	−0.292^*^ (−0.370, −0.209)	−0.239^*^ (−0.320, −0.155)	−0.270^*^ (−0.349, −0.187)	−0.307^*^ (−0.383, −0.225)	1		
Work limitations	−0.243^*^ (−0.323, −0.158)	−0.234^*^ (−0.315, −0.150)	−0.175^*^ (−0.259, −0.089)	−0.232^*^ (−0.313, −0.148)	−0.202^*^ (−0.284, 0.117)	−0.229^*^ (−0.310, −0.144)	−0.242^*^ (−0.323, −0.158)	−0.197^*^ (−0.280, −0.111)	−0.223^*^ (−0.304, −0.138)	−0.257^*^ (−0.336, −0.173)	0.858^*^ (0.883, 0.880)	1	
Work Energy	−0.238^*^ (−0.318, −0.153)	−0.211^*^ (−0.293, −0.126)	−0.184^*^ (−0.267, −0.098)	−0.203^*^ (−0.285, −0.117)	−0.219^*^ (−0.301, −0.134)	−0.213^*^ (−0.295, −0.128)	−0.146^*^ (−0.230, −0.059)	−0.122^*^ (−0.207, −0.035)	−0.137^*^ (−0.221, −0.050)	−0.149^*^ (−0.234, −0.063)	0.451^*^ (0.378, 0.518)	−0.071 (−0.157, 0.017)	1

^*^P < 0.05.

### 3.7 Covariance test

A test of covariance was conducted with presenteeism as the dependent variable and four variables with statistically significant differences in general demographic information for presenteeism as the independent variables, as well as professional self-concept and perceived social support, and found that the VIF for Satisfaction with Effort and Compensation under “Neutral”, “Satisfied” had a VIF >5 (Table 7). Therefore subsequent stratified regression analysis was conducted to exclude Satisfaction with Effort and Compensation.

**Table 7:** Co-linearity test.

**Variable**	**Intra-class predictor variables**	**Tolerance**	**VIF**
Department	Surgery	0.732	1.366
	Operating room	0.784	1.276
	Others	0.805	1.242
Did your job require night shifts in the past year?	No	0.915	1.093
Evaluation of current work intensity	Relatively high	0.485	2.062
	Moderate	0.472	2.121
	Relatively low	0.884	1.131
	Very low	0.967	1.034
Satisfaction with effort and compensation	Dissatisfied	0.270	3.710
	Neutral	0.132	7.556
	Satisfied	0.150	6.664
	Very satisfied	0.456	2.194
Professional self-concept scale		0.685	1.460
Perceived social support scale		0.699	1.430

### 3.8 Hierarchical regression analysis of presenteeism

**Figure 2:**
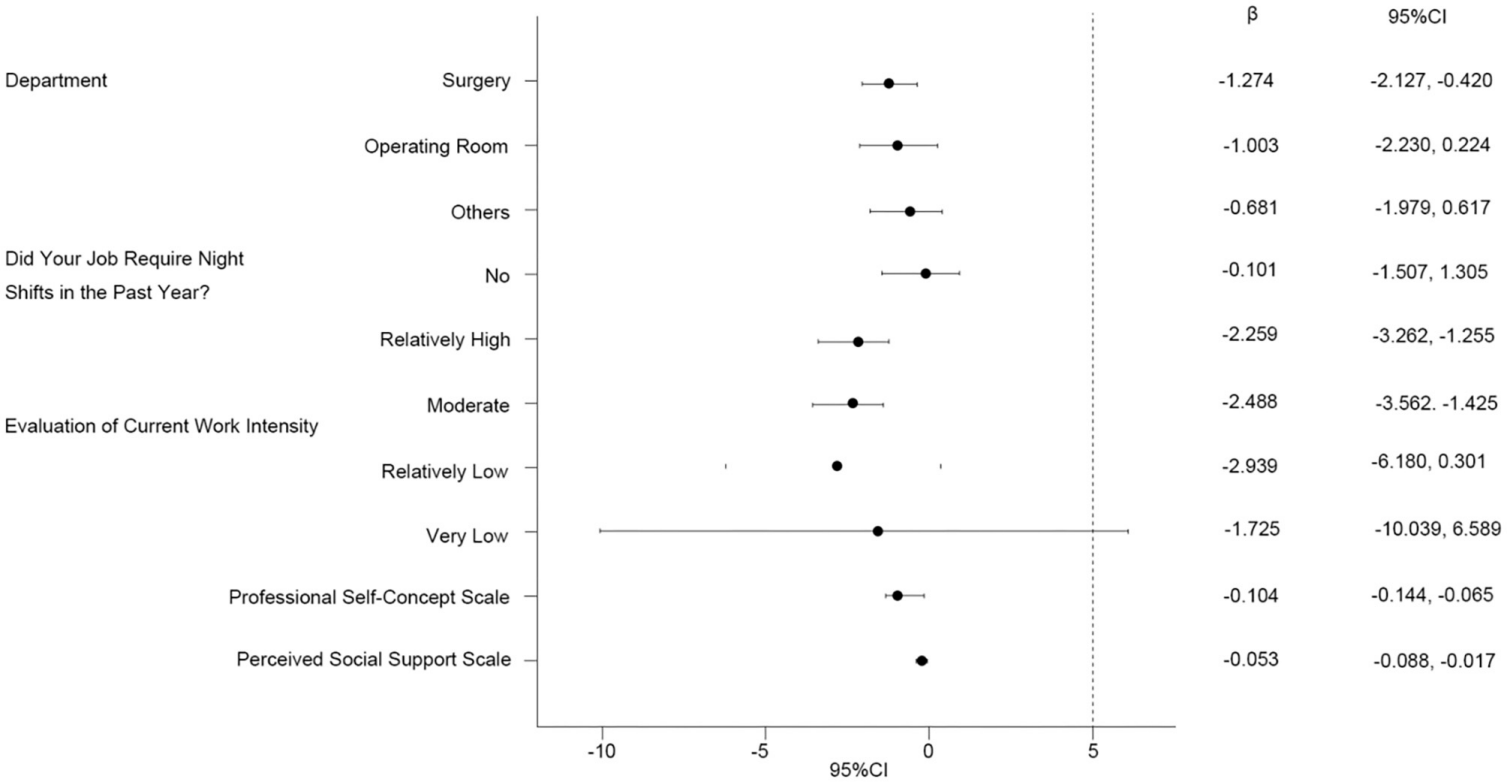
Impact of each subgroup on presenteeism.

Table 8 shows the assignment of values to the independent variables. Hierarchical multiple regression analysis showed that in Model 1, surgery, operating room, and work intensity evaluation of “relatively high” and “moderate” had obvious predictive effects on presenteeism (*P* < 0.05, Table 9). In Model 2, surgery, work intensity evaluation of “relatively high” and “moderate”, and professional self-concept had obvious predictive effects on presenteeism (*P* < 0.01, Table 9). In Model 3, surgery, operating room, work intensity evaluatio n of “relatively high” and “moderate”, professional self-concept, and perceived social support had obvious predictive effects on presenteeism (*P* < 0.05, Table 9). The *R*^2^ of Model 1, Model 2, and Model 3 were 6.6%, 16.1%, and 17.4% respectively. The 95% confidence interval for Δ*R*^2^ for Model 1 to Model 2 is (0.051, 0.139), and the 95% confidence interval for Δ*R*^2^ for Model 2 to Model 3 is (−0.003, 0.031). Figure 2 shows the effect of surgery, OR and work intensity ratings of “relatively high” and “moderate”, professional self-concept, perceived social support on presenteeism.

**Table 8:** Specific assignments of independent variables and related dummy variables.

**Variables**	**Specific assignment of independent variables and related dummy variables**
Department	Internal medicine (Z1 = 0, Z2 = 0, Z3 = 0, Z4 = 0) Surgery (Z1 = 0, Z2 = 1, Z3 = 0, Z4 = 0) Operating room (Z1 = 0, Z2 = 0, Z3 = 1, Z4 = 0) Others (Z1 = 0, Z2 = 0, Z3 = 0, Z4 = 1)
Did your job require night shifts in the past year?	Yes = 1, No = 2
Evaluation of current work intensity	Very high(Z1 = 0, Z2 = 0, Z3 = 0, Z4 = 0, Z5 = 0) Relatively high (Z1 = 0, Z2 = 1, Z3 = 0, Z4 =, Z5 = 0) Moderate (Z1 = 0, Z2 = 0, Z3 = 1, Z4 = 0, Z5 = 0) Relatively low (Z1 = 0, Z2 = 0, Z3 = 0, Z4 = 1, Z5 = 0) Very low (Z1 = 0, Z2 = 0, Z3 = 0, Z4 = 0, Z5 = 1)
Professional self-concept	Raw value input
Social support	Raw value input

**Table 9:** Multiple linear hierarchical regression analysis of factors influencing recessive presenteeism.

**Variables**	**Intra-class predictor variables**	**Model 1**			**Model 2**			**Model 3**		
		β	**95% CI**	***P***	β	**95% CI**	***P***	β	**95% CI**	***P***
Department	Surgery	−1.237	−2.145, −0.330	0.008	−1.254	−2.114, −0.395	0.004	−1.274	−2.127, −0.420	0.004
	Operating room	−1.516	−2.813, −0.219	0.022	−1.008	−2.244, 0.228	0.11	−1.003	−2.230, 0.224	0.109
	Others	−0.844	−2.224, 0.535	0.230	−0.755	−2.062, 0.552	0.257	−0.681	−1.979, 0.617	0.303
Did your job require night shifts in the past year?	No	−1.388	−2.845, 0.069	0.062	−0.187	−1.602, 1.229	0.795	−0.101	−1.507, 1.305	0.888
Evaluation of current work intensity	Relatively high	−2.465	−3.529, −1.400	0.001	−2.349	−3.359, −1.340	0.001	−2.259	−3.262, −1.255	0.001
	Moderate	−2.716	−3.853, −1.579	0.001	−2.625	−3.702, −1.547	0.001	−2.488	−3.562. −1.425	0.001
	Relatively low	−3.185	−6.627, 0.257	0.070	−3.153	−6.415, 0.109	0.058	−2.939	−6.180, 0.301	0.075
	Very low	−1.330	−10.149, 7.488	0767	−2.480	−10.841, 5.882	0.560	−1.725	−10.039, 6.589	0.684
Professional self-concept scale					−0.133	−0.168, −0.099	0.001	−0.104	−0.144, −0.065	0.001
Perceived social support scale								−0.053	−0.088, −0.017	0.004
Regression model	*F*	5.415		0.001	11.704		0.001	11.550		0.001
	*R*	0.284			0.420			0.436		
	*R^2^*	0.081			0.176			0.190		
	Adjusted *R^2^*	0.066			0.161			0.174		
	Δ*R*^2^	–			0.095			0.014		

### 3.9 Modeling of mediating effects

**Figure 3:**
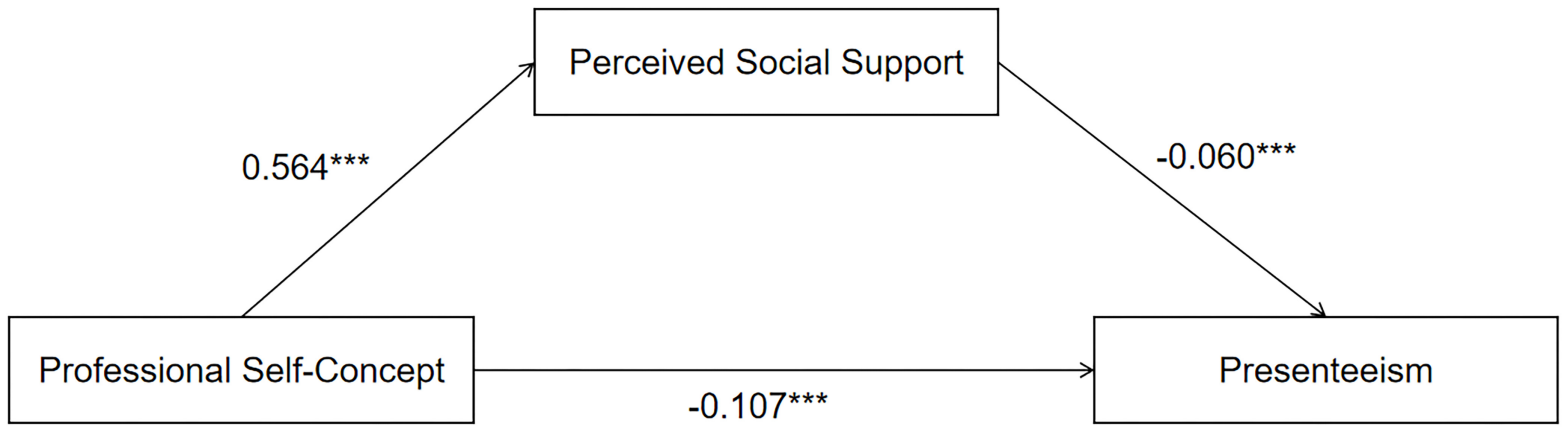
A model of the mediating effect of perceived social support between professional self-concept and presenteeism. ***P < 0.05.

The model indicated that professional self-concept had a significant direct effect on perceived social support with a value of 0.564 (95% confidence interval of 0.481 to 0.647) and on presenteeism with a value of −0.107 (95% confidence interval of −0.147 to −0.068). The direct effect value of perceived social support on presenteeism was −0.060 (95% confidence interval of −0.096 to −0.024). The indirect effect of professional self-concept on presenteeism through perceived social support was −0.034 (95% confidence interval −0.058 to −0.011), which suggests that perceived social support partially mediates the relationship between professional self-concept and presenteeism (Table 10, Figure 3).

**Table 10:** Total, direct, and indirect effects of professional self-concept on presenteeism.

**Effect**	**Paths**	**Effect value**	**Standardized estimates**	***P***	**95%CI**
Direct	Professional self-concept → Perceived social support	0.564	0.042	0.000	0.481, 0.647
	Professional self-concept → Presenteeism	−0.107	0.020	0.000	−0.147, −0.068
	Perceived social support → Presenteeism	−0.060	0.018	0.000	−0.096, −0.024
Indirect	Professional self-concept → Perceived social support → Presenteeism	−0.034	0.012	0.000	−0.058, −0.011
Total	Professional self-concept → Presenteeism	−0.141	0.018	0.000	−0.176, −0.106

## 4 Discussion

Presenteeism is a well-recognized public health concern due to its close link to health outcomes and has been studied across various occupational groups (25). Nurses represent a typical population experiencing high rates of presenteeism (26). A meta-analysis of 28 studies from 14 countries reported a nearly 50% overall prevalence of presenteeism among nurses (6). This highlights the widespread nature of the issue within the nursing profession. For example, a cross-sectional survey in Switzerland found an 84.3% prevalence among nurses and midwives (27). Similarly, in China, Li et al. (28) surveyed 3,491 nurses across 14 hospitals in Shandong Province and identified a 70.6% prevalence rate. These findings underscore the global nature of presenteeism among nurses. Growing evidence highlights the detrimental effects of presenteeism, which outweigh its benefits. It not only impairs nurses‘ physical and mental health but also increases patient safety risks, reduces care quality, and may result in greater economic losses than absenteeism (26, 29, 30). This study examined the relationship between professional self-concept, perceived social support, and presenteeism among Chinese nurses. Therefore, understanding nurses' current status and needs can help address presenteeism among nursing professionals, reduce absenteeism disguised as presence, and play a crucial role in enhancing clinical care quality, stabilizing the nursing workforce, and minimizing productivity losses.

The total presenteeism score in this study was 15.14 ± 4.58 points, which was lower than the 21.00 ± 4.3 reported by Shdaifat (31). The total career self-concept score in this study was 86.79 ± 11.01 (moderate), which was higher than the results of Cho et al.'s (13) study in Korea (69.92 ± 7.19). This discrepancy may stem from cross-national cultural differences in language, educational systems, or cultural values. Our demographic analysis of presenteeism identified high-risk groups among nurses with heavy workloads, significant economic pressures, and insufficient support. In China, older nurses are more likely to have permanent positions or long-term contracts that guarantee a stable income and predictable retirement benefits. This institutional protection reduces the financial pressure to work during periods of illness. In contrast, younger nurses, especially those on short-term or agency contracts, may face performance-based pay and the threat of non-renewal, making “presenteeism” a rational survival strategy (32). Specifically, internal medicine nurses exhibited significantly higher presenteeism scores than surgical or operating room nurses. Nurses working night shifts or reporting “very high” workloads showed increased presenteeism due to physiological fatigue and diminished focus. Psychological and economic factors were particularly salient: nurses expressing “very dissatisfaction” with compensation or facing “severe financial strain” experienced reduced productivity due to burnout or financial distraction. Conversely, those satisfied with salaries and in stable economic conditions demonstrated lower presenteeism risks due to positive mindsets (33, 34).

Studies show that examining nurses' professional self-concept advances research on job satisfaction and occupational stress. A positive professional self-concept in nursing fosters higher occupational identity, job satisfaction, and reduces burnout and turnover intentions. Nurses with stronger professional self-concept exhibit greater professional identity and belonging, facilitating career adaptation and growth (12, 35, 36). This study reveals a “high at both ends, low in the middle” pattern in perceived social support differences. Younger nurses (< 25) may receive more mentorship, while those ≥40 gain respect through seniority. Nurses aged 35–39 reported lower perceived social support, possibly due to mid-career stressors like promotion barriers and work-family conflict, though this remains speculative without interaction testing. Occupational factors also matter: administrative roles and permanent positions offer greater resources, while night shifts and long hours limit social interaction. Economic status influences support too-higher income and financial comfort enhance perceived support, whereas dissatisfaction with pay or financial strain may reduce it, creating a negative cycle (37–39). However, it is important to note that this interpretation remains speculative, as the current study has not yet conducted statistical tests for interaction effects (age × social support) to empirically validate this relationship.

The study findings revealed a significant negative correlation between total perceived social support scores and presenteeism scores. Specifically, dimensions such as family support, friend support, and other forms of support (e.g., from colleagues, supervisors) all showed significant negative correlations with presenteeism. This suggests that perceived social support is predictive of lower levels of presenteeism behaviors in nurses. Nurses with strong family support can obtain adequate rest and care outside of work, maintaining physical and mental well-being for their job. Those with robust friend support gain emotional encouragement and advice during work pressures, enhancing their stress-coping abilities. Other forms of support, such as assistance from colleagues and supervisors, help nurses address practical challenges, improve job satisfaction, and ultimately reduce presenteeism (16, 40).

Hierarchical regression analysis identified surgical departments as a consistent predictor of presenteeism. High-risk environments, frequent emergencies, and prolonged standing in surgical units may lead to chronic fatigue and mental detachment, increasing presenteeism (e.g., reduced focus) (41). The positive link between perceived workload and presenteeism suggests compensatory behaviors (e.g., task delays) to manage overload. Self-rated workload measures, however, may introduce bias; future studies should incorporate objective metrics (e.g., surgeries per hour) (42). The persistent significance of management skills (Models 2–3) highlights self-efficacy's role: strong planners reduce disorganization-related presenteeism. Models 1, 2, and 3 explained 6.6%, 16.1%, and 17.4% of presenteeism variance, respectively. Adding professional self-concept (particularly management skills) increased explanatory power from 6.6% to 16.1%. The results of the regression model in this study showed limited explanatory power. And the omission of relevant variables that were not measured in this study may have affected the variance explained by the model. For example, in a study by Demerouti et al. (43), the inclusion of variables such as job resources (e.g., job autonomy, social support) and individual resources (e.g., optimism, self-esteem) explained about 25% of the variance in presenteeism. In addition, Bergström et al. (44) showed that the variables of work environment, health status, and job stress explained about 30% of the variance in presenteeism. Another study conducted by Aronsson and Gustafsson (45) showed that the variables of job insecurity, sense of organizational fairness, and health status significantly predicted the occurrence of presenteeism. These studies suggest that presenteeism is a complex phenomenon influenced by multiple factors. The limited inclusion of variables in this study may have limited the model's ability to capture the true complexity of the phenomenon. Therefore, future studies should consider incorporating additional variables that may contribute to the variability of presenteeism.

Our study found that perceived social support was a mediator between professional self-concept and presenteeism, and that perceived social support and professional self-concept were negatively related with presenteeism. Lin et al. (46) surveyed 468 registered nurses in Sichuan Province, China at Level IIIA and showed that presenteeism was negatively related to perceived social support. The above findings are consistent with our findings. However, the relationship between professional self-concept and presenteeism has not been studied yet. This study on perceived social support playing a role in professional self-concept and presenteeism has important implications for nurses. This can be explained by COR, where social support can be considered as a psychological resource (47). And professional self-concept can be considered as an important internal energy resource for nurses (48). This reflects their recognition and confidence in their professional competence and professional value. This positive self-concept is associated with more efficient coping with work challenges among nurses and may predict lower resource consumption linked to self-doubt or competence anxiety, thus showing an inverse relationship with presenteeism (33). The role of perceived social support as a mediating variable in this study further supports the logic of “resource interaction” in COR theory: nurses with a strong professional self-concept are more likely to establish and perceive social support around them (i.e., intrinsic resources facilitate access to extrinsic resources), and such activated resources of social support will further enhance their ability to cope with stress. However, frequent presenteeism may likewise lead to a gradual weakening of self-concept. This finding provides implications for nursing management practice: reducing nurse presentism should not only focus on adjustments at the individual level, but should also be considered from a resource conservation perspective. We should enhance nurses' professional self-concept through professional training and professional identity education (strengthening internal resources), and on the other hand, we should build a perfect social support system (supplementing external resources), such as optimizing the teamwork mechanism, establishing a leadership care system, improving family-work balance support, and so on. On the other hand, it is necessary to build a perfect social support system (supplementing external resources), such as optimizing the teamwork mechanism, establishing a leadership care system, and improving the family-work balance support. Through the synergistic protection of internal and external resources, we can more effectively reduce presenteeism and maintain the physical and mental health of nurses and the quality of nursing work.

## 5 Conclusion

This study found that nurses' occupational self-concept, perceived social support and presenteeism are negatively correlated, and that social support partially mediates the relationship between occupational self-concept and presenteeism. Based on this finding, the following specific and evidence-supported policy recommendations are put forward for hospital administrators: establish a mentorship program for early-career nurses, and help early-career nurses understand their occupational roles, abilities, and values more clearly by providing them with guidance through experienced nurses. For early career nurses, a mentorship program should be established to help early career nurses have a clearer understanding of their professional roles, abilities and values through the guidance of experienced nurses, so that they can strengthen their professional self-concept and reduce the likelihood of attendanceism. The role of social support in mitigating attendanceism; the assessment of professional self-concept and perceived social support is incorporated into the daily management system of nurses, so as to regularly understand the status of nurses in these two aspects, and for nurses with deficiencies, targeted interventions are taken in a timely manner, such as carrying out career development training, providing psychological counseling, etc., so as to reduce the phenomenon of attendanceism from an overall perspective.

## 6 Limitations

While this study offers new insights for nursing management, limitations exist. This study has limitations in regional sampling. Differences exist in medical resource allocation, nursing work environments, work pressures and career development opportunities across regions and between medical institutions of different levels, which may affect nurses' professional self-concept, social support and presenteeism. Since samples only come from tertiary hospitals in Guangdong Province, whose nurses differ greatly from those in primary medical institutions or rural areas in work scenarios and professional experiences, the conclusions on the correlation between the three can only reflect the situation of nurses in such hospitals and cannot be directly extended to others, thus limiting the universality of the research conclusions to a certain extent. Therefore, the follow-up study will consider expanding the geographic scope to include samples of nurses from more provinces and different levels of healthcare institutions (including first- and second-level hospitals and healthcare institutions in rural areas, etc.), to further validate the applicability of the findings of the present study by comparatively analyzing the relationship between occupational self-concept, social support, and presenteeism among the different groups, and to enhance the generalizability and generalizable value of the findings.

This study adopted a cross-sectional design. It only collected data on nurses‘ professional self-concept, social support and presenteeism at a specific time point, and then analyzed the associations among the three. It should be made clear that such associations only reflect the coexistence relationship between variables and do not equal causal relationships. Because the cross-sectional design could not track the dynamic changes of variables over time, nor determine the order of variables, it was impossible to infer whether changes in nurses' professional self-concept or social support led to changes in presenteeism, or whether the status of presenteeism affected their professional self-concept or the social support they received. In view of this, future studies can consider adopting a longitudinal design. By tracking the same group of nurses for a long time and collecting data at different time points, they can explore the possible causal relationships among the three more deeply, so as to provide a more solid theoretical basis for the formulation of relevant intervention measures.

## Data Availability

The raw data supporting the conclusions of this article will be made available by the authors, without undue reservation.
